# Whole-Genome Sequencing Analysis of Quorum-Sensing Aeromonas hydrophila Strain M023 from Freshwater

**DOI:** 10.1128/genomeA.01548-14

**Published:** 2015-02-19

**Authors:** Wen-Si Tan, Wai-Fong Yin, Chien-Yi Chang, Kok-Gan Chan

**Affiliations:** a Division of Genetics and Molecular Biology, Institute of Biological Sciences, Faculty of Science, University of Malaya, Kuala Lumpur, Malaysia; b Interdisciplinary Computing and Complex BioSystems (ICOS) Research Group, School of Computing Science, Newcastle University, Newcastle upon Tyne, United Kingdom; c Centre for Bacterial Cell Biology, Medical School, Newcastle University, Newcastle upon Tyne, United Kingdom

## Abstract

Aeromonas hydrophila is a well-known waterborne pathogen that recently was found to infect humans. Here, we report the draft genome of a freshwater isolate from a Malaysian waterfall, A. hydrophila strain M023, which portrays *N*-acylhomoserine lactone-dependent quorum sensing.

## GENOME ANNOUNCEMENT

Bacteria communicate to modulate different physiological activities (1). This process is termed quorum sensing (QS), or bacteria cell-cell communication, which relies on the production and response of small diffusible molecules in relation to population density (2). Aeromonas hydrophila is ubiquitous in water medium and tends to be pathogenic to humans by causing gastroenteritis (3, 4). A. hydrophila can also cause major economic loss in aquaculture (5). In this study, A. hydrophila strain M023 was isolated from a waterfall sample. The whole-genome sequencing was conducted for better understanding of the quorum-sensing system of this strain.

Genomic DNA of strain M023 was extracted by using a MasterPure DNA purification kit (Epicentre, Inc., Madison, WI, USA) with its quality checked by NanoDrop spectrophotometer (Thermo Scientific, Waltham, MA, USA) and a Qubit version 2.0 fluorometer (Life Technologies, Carlsbad, CA, USA). Whole-genome shotgun sequencing of the purified DNA was then performed with the Illumina MiSeq platform (Illumina, Inc., CA, USA), which generated 5,031,085 paired-end reads. Next, the sequences were trimmed and *de novo* assembled with CLC Genomic Workbench version 5.1 (CLC Bio, Denmark). The assembly of trimmed reads (817,464 quality reads) generated a total of 183 contigs and an *N*_50_ of approximately 67,516.

The draft genome of the strain M023 isolate comprises 4,914,534 bases with an average coverage of 30.5-fold with 61% of G+C content. Next, gene prediction was conducted with the prokaryote gene prediction algorithm by using Prodigal version 2.60 (6), while the amount of tRNAs and rRNAs were predicted using tRNAscan SE version 1.21 (7) and RNAmmer (8), respectively. The strain was then annotated using RAST (9). The open reading frames of strain M023 were predicted at a total of 4,338. There are 6 copies of 5S rRNAs and a single copy each of 23S rRNA and 16S rRNA, while a total of 101 tRNAs were found in the genomes of strain M023.

From the annotation results, the *luxI* and *luxR* homologues of strain M023 were predicted to be located at contig 9. The whole-genome sequence allows better understanding of the genetic makeup of A. hydrophila to determine the link between quorum sensing and pathogenicity and production of virulence factors (10, 11).

### Nucleotide sequence accession numbers.

This whole-genome shotgun project has been deposited at DDBJ/EMBL/GenBank under the accession number JSWA00000000. The version described in this paper is the first version, JSWA01000000.
